# Deep compartment models: A deep learning approach for the reliable prediction of time‐series data in pharmacokinetic modeling

**DOI:** 10.1002/psp4.12808

**Published:** 2022-05-27

**Authors:** Alexander Janssen, Frank W. G. Leebeek, Marjon H. Cnossen, Ron A. A. Mathôt, for the OPTI‐CLOT study group and SYMPHONY consortium, K. Fijnvandraat, M. Coppens, K. Meijer, S. E. M. Schols, H. C. J. Eikenboom, R. E. G. Schutgens, E. A. M. Beckers, P. Ypma, M. J. H. A. Kruip, S. Polinder, R. Y. J. Tamminga, P. Brons, K. Fischer, K. P. M. van Galen, L. Nieuwenhuizen, M. H. E. Driessens, I. van Vliet, J. Lock, H. C. A. M. Hazendonk, I. van Moort, J. M. Heijdra, M. H. J. Goedhart, W. Al Arashi, T. Preijers, N. C. B. de Jager, L. H. Bukkems, M. E. Cloesmeijer, P. W. Collins, R. Liesner, P. Chowdary, C. M. Millar, D. Hart, D. Keeling

**Affiliations:** ^1^ Department of Clinical Pharmacology, Hospital Pharmacy Amsterdam University Medical Center Amsterdam The Netherlands; ^2^ Department of Hematology Erasmus University Medical Center Rotterdam The Netherlands; ^3^ Department of Pediatric Hematology Erasmus University Medical Center‐Sophia Children's Hospital Rotterdam The Netherlands

## Abstract

Nonlinear mixed effect (NLME) models are the gold standard for the analysis of patient response following drug exposure. However, these types of models are complex and time‐consuming to develop. There is great interest in the adoption of machine‐learning methods, but most implementations cannot be reliably extrapolated to treatment strategies outside of the training data. In order to solve this problem, we propose the deep compartment model (DCM), a combination of neural networks and ordinary differential equations. Using simulated datasets of different sizes, we show that our model remains accurate when training on small data sets. Furthermore, using a real‐world data set of patients with hemophilia A receiving factor VIII concentrate while undergoing surgery, we show that our model more accurately predicts a priori drug concentrations compared to a previous NLME model. In addition, we show that our model correctly describes the changing drug concentration over time. By adopting pharmacokinetic principles, the DCM allows for simulation of different treatment strategies and enables therapeutic drug monitoring.

**Study Highlights**

**WHAT IS THE CURRENT KNOWLEDGE ON THE TOPIC?**

Current implementations of machine learning (ML) in pharmacometrics cannot reliably be extrapolated to timepoints and treatment outside of the training data.

**WHAT QUESTION DID THIS STUDY ADDRESS?**

Can we develop a ML model that can be used to accurately predict drug concentrations by constraining the solution using differential equations?

**WHAT DOES THIS STUDY ADD TO OUR KNOWLEDGE?**

The proposed deep compartment model does not require large data sets, can be individualized to unique treatment schedules of patients, and is highly accurate on unseen data.

**HOW MIGHT THIS CHANGE DRUG DISCOVERY, DEVELOPMENT, AND/OR THERAPEUTICS?**

Our study opens the door for the reliable use of ML for many applications in the field of pharmacometrics. The method is extremely fast, sharply reducing the time spent developing complex mathematical models. Because we are explicitly adding known constraints to our model, we reduce the need for large data sets which is often a limitation for the implementation of ML in the field of pharmacometrics.

## INTRODUCTION

There is much interest in the adoption of machine learning (ML) in the field of pharmacometrics. Implementation of covariates in population pharmacokinetic (PK) models can be very complex, and might benefit from the automatic learning capabilities of ML algorithms. Previous studies have examined the accuracy of such models for predicting drug concentrations.^1^, ^2^, ^3^ Although these studies report similar or improved accuracy compared to nonlinear mixed effect (NLME) models, which are widely considered to be the gold standard in the field, none of these models allow for practical use. For example, most of the proposed ML models have only been trained to predict drug concentrations at specific timepoints. Extrapolating from these timepoints can lead to highly inaccurate results. In addition, dosing and timing information is often a direct input to the model, even though we are uncertain that they will be interpreted as such. As a result, trust in the ML algorithm is low because we do not understand the translation from covariates to drug concentrations. A simple way to overcome these issues is to constrain the solution space to satisfy knowledge about drug dynamics. This involves using an ML model to predict the latent parameters z of another function, such as the one compartment model:

(1) $$C\left(t,D\right)=\frac{D}{V_{d}}\cdot \exp\left(-k_{e}t\right),z\in \left\{V_{d},k_{e}\right\}$$

Here, the elimination rate constant ($k_{e}$) and the distribution volume ($V_{d}$) of the drug are estimated by an ML model, whereas dose D and time since dose t can be supplied directly to $C\left(t,D\right)$. If the drug is eliminated at a constant concentration‐dependent rate, we can thus reliably extrapolate to different timepoints or doses. Unfortunately, for most drugs, this assumption does not hold, and as soon as the complexity of the compartment model or dosing schedule increases, no simple closed form solution exists.

A recent paper by Chen et al.^4^ reports on an automatic differentiation method for calculating the gradient of an ordinary differential equation (ODE) solution with respect to its inputs. This means that methods relying on automatic differentiation for gradient calculations, such as neural networks, can be constrained based on ODEs. Because we can represent any compartment model using a system of ODEs, this opens the door for a reliable use of ML algorithms in the field of pharmacometrics. In addition, interventions (such as drug doses) can be defined to perturb the ODE system at specific timepoints, allowing for the differentiation of the solution with respect to individual treatment schedules.

In this study, we present the deep compartment model (DCM). In a DCM, a neural network is used to predict the latent parameters of a system of ODEs representing a compartment model. This technique allows for a full model‐based approach which automatically implements covariates in PK models. We will test the accuracy of this model for predicting drug concentrations using simulated data sets of different sizes. In addition, we will compare its accuracy to an NLME model on real‐world data of patients with hemophilia A receiving standard half‐life (SHL) factor VIII (FVIII) concentrate while undergoing surgery. Both models will be fit on a retrospective data set,^5^ and will be validated on data collected during the OPTI‐CLOT randomized controlled trial.^6^

## RELATED WORK

Brier et al. discussed a comparison of steady‐state peak and trough gentamicin concentrations predictions made by a neural network and NLME model.^1^ The neural network predicted peak gentamicin concentrations between 2.5 and 6.0 μg/ml with lower bias compared to the NLME model. However, when extrapolating to samples which were outside of this range (and not in the training set) the NLME model was more accurate. This indicated that using ML algorithms as is likely results in problems with respect to extrapolating to unseen data.

Lai et al. introduce an implementation of neural networks (and regression splines) in the likelihood function for a nonparametric estimation of covariate effects in population PK models.^7^ The neural network was used to directly learn the relationship between covariates and the PK parameters of a one‐compartment model. They show how the neural network is able to accurately represent nonlinear effect of covariates. However, the approach focuses on the use of compartmental models with a closed‐form solution and is difficult to extend to more complex models.

Finally, Lu et al. reported on the deep‐learning‐based approach which utilizes a neural ODE^4^ to handle time and dose irregularities.^8^ A recurrent neural network encoder is used to learn the initial state for an ODE solver. The solver translates this state based on the current time interval between doses into a latent variable space z. Finally, a decoder is used to translate samples from z to the concentration predictions. The authors show how this approach can be used to correctly extrapolate to treatment schedules not seen during training, in contrast to other ML‐based methods. However, a possible issue is its inherent reliance on black box methods for estimation. It is difficult to understand what the latent variables z represent, how the neural ODE produces them, and finally how the decoder relates them to the observations.

Results from the above papers indicate how using time and dose as direct inputs to ML models will likely lead to poor extrapolation to samples outside of the training data. This is eloquently shown by Lu et al., where such models still predict drug exposure even when the given dose is set to zero.^8^ In this work, neural networks are used to predict parameters for an ODE (similar to NLME models), which makes it easier to implement complex compartment models and dosing schedules. The proposed architecture is relatively simple compared to the NeuralODE.^8^ The latent variables z predicted by the neural network now represent PK parameters, which are more interpretable and can be compared to previous results.

## METHODS

### Problem definition

We consider a dataset of n patients with d observed covariates $x_{i}\in X^{n\times d}$, $i\in \left\{1\ldots n\right\}$; and corresponding drug concentration measurements $y_{i}\in {\mathbb{R}}_{+}^{k}$ for k measurements in time window $t\in \left[0,T\right]$. The number of measurements may differ between patients. For each patient i, we can define a set of clinical interventions $I_{i}$, which, for example, contains information of drug doses given at specific timepoints. In classical PK modeling, we can represent the dynamics of this drug using a system of ODEs $A\left(t,z,I\right)$ with p latent parameters $z\in {\mathbb{R}}_{+}^{p}$ (aptly named the PK parameters). We often assume that the information in X is insufficient to completely describe the interindividual variation (IIV) in the concentration measurements, so our goal is to predict the typical or population predicted concentrations $E\left[y_{i}\right]$. The corresponding typical PK parameters $\zeta_{i}$ for each patient are predicted directly from the covariates using a set of functions $f_{\theta }$ so that:

(2) $$\zeta_{i}=f_{\theta }\left(x_{i}\right).$$

The algebraic form of $f_{\theta }$ has to be specified but its parameters θ can be estimated from data. In many cases, prior knowledge is present for choosing an appropriate compartment model, but not $f_{\theta }$. As a result, implementations of $f_{\theta }$ can be suboptimal, resulting in lower accuracy of $E\left[y_{i}\right]$. To combat this issue, NLME models introduce two random variables: one describing the IIV: $\eta ∼N\left(0,\Omega \right)$, and one describing the residual variability: $\epsilon ∼N\left(0,\sum \right)$. η is used to transform ζ to obtain a distribution of z which describes the residual IIV in the population:

(3) $$z=\zeta \cdot \exp\left(\eta \right)$$

Here, we have depicted a commonly used transformation of ζ which results in a log normally distributed random variable z. NLME models predict a set of parameters $\Theta =\left\{\theta ,\Omega ,\sum \right\}$ and produces a maximum a posteriori estimate of η which maximizes $p\left(\eta |y_{i},\Theta \right)$. A downside of this approach is the requirement of sufficient measurements in $y_{i}$, especially when T is large. Because the a priori predicted $E\left[y_{i}\right]$ can be inaccurate, we often need to generate a PK profile for new patients. This can be perceived as an additional burden for the patient, especially when measurements need to be taken over the span of multiple days.

### Deep compartment model

In order to improve the prediction of ζ we developed the DCM. Here, a neural network $\phi_{w}$ with weights w is used to predict the latent parameters of a compartment model based on $I_{i}$. Because $\phi_{w}$ directly predicts ζ instead of $y_{i}$, we can better interpret its output. The neural network learns to represent ζ from a latent z in a nonparametric manner. When we assume that each concentration measurement $y_{\mathrm{ij}}$ is drawn i.i.d. from a Gaussian distribution with mean $\mu_{\mathrm{ij}}$ and variance $\sigma^{2}$ so that $y_{\mathrm{ij}}=\mu_{\mathrm{ij}}+\epsilon_{\mathrm{ij}}$, $\epsilon_{\mathrm{ij}}∼N\left(0,\sigma^{2}\right)$; we can find the optimal weights $w^{*}$ by minimizing the mean squared error (MSE) objective function:

(4) $$w^{*}=\min_{w}\;L\left(X\right)=\frac{1}{n}\sum_{i=1}^{n}{\left(y_{i}-A\left(t_{i},\phi_{w}\left(x_{i}\right),I_{i}\right)\right)}^{2}$$

The DCM model was developed in the Julia programming language (Julia Computing, Inc., version 1.6.0). Dosing events in $I_{i}$ were implemented as time‐based callbacks to the ODE solver. These callbacks affected the rate of drug flowing into the central compartment. Consequently, bolus doses were converted to short duration infusions with a fixed duration of 1 minute and rate D·60 IU/h. Model covariates were normalized between zero and one using minimum‐maximum normalization. Two variants of the DCM were developed. The first directly outputs ζ in the final layer, using a softplus activation function to ensure ζ≥0. The second can be passed a set of initialization parameters $\zeta_{0}$. In the latter case, the final layer of $\phi_{w}$ has the following form:

(5) $$l_{n}=\zeta_{0}⊙\left(\pi \left(l_{L-1}\right)+\vec{1}\right)$$

Here, L denotes the number of layers l in $\phi_{w}$, ⊙ indicates the Hadamard product, $\pi \left(\cdot \right)$ is the CELU activation function with α<1, ^9^ and $\vec{1}$ is a vector of ones of length p. In this case, the model learns the deviation from $\zeta_{0}$ based on $x_{i}$. The CELU activation function acts as an implicit constraint to penalize the gradient of $l_{n-1}$ as it reaches 1−α, preventing ζ to be zero. The “standard” DCM can be used in cases where measurement data is rich, whereas the DCM with initialization can help to improve parameter predictions when data are sparse.

In this paper, we have used a basic neural network encoder structure in order to reduce the number of parameters in the model. The model contained two fully connected hidden layers: the first had 64 neurons, and the second had 16 neurons. The swish activation function was used for the hidden layers.^10^ The output layer contained four neurons representing the PK parameters. No optimization of model architecture was performed. The ADAM optimizer was used for updating neural network weights with a learning rate of 1e‐3.^11^

All relevant code and results will be made available for public access at https://github.com/Janssena/DeepCompartmentModels.jl at the time of publication.

### Simulation experiment

We simulated a data set of 500 patients based on a previously published NLME model.^5^ This model was developed using retrospective data from 119 patients with hemophilia A treated with an SHL FVIII concentrate perioperatively. This model predicted ζ based on patient weight, age, blood group, and surgical risk score. A two‐compartment model with clearance (CL), central volume of distribution (V1), intercompartmental clearance (Q), and peripheral volume (V2) parameters was used.

The goal of our simulation was to evaluate the accuracy of the DCM in sparse and dense data scenarios. For each patient, we simulated a single intravenous dose of 25–50 IU kg^−1^ (rounded to nearest multiple of 250) of SHL FVIII concentrate at *t* = 0. Typical PK parameters were calculated based on samples from covariate distributions fit to the original dataset. FVIII levels were simulated based on these PK parameters and collected at *t* = 0.5 h and every hour until *t* = 48 h. Average simulated FVIII peak level was 0.89 IU ml^−1^ (0.43–1.31), and average trough level at *t* = 48 h was 0.09 IU ml^−1^ (0.01–0.21). Gaussian noise (σ=0.05) was added to produce training measurements. Any resulting negative concentrations were fixed to zero. Multiple sets of measurements were collected to evaluate an extremely limited (*t* = 24), limited (*t* = 8, 30), routine (*t* = 4, 24, 48), and extensive (*t* = 0.5, 4, 12, 24, 36, and 48) sampling strategy.^12^ The DCM was trained on 20, 60, or 120 patients representing datasets of low, medium, and large size, respectively. Corresponding test sets contained the remaining 480, 440, or 380 patients. Models were trained until MSE stopped improving. Both a standard DCM and DCM with initialization were fit for all scenarios. A reasonable set of initialization parameters $\zeta_{0}=\left[150,2500,150,2000\right]$ was used for CL (ml/h), V1 (ml), *Q* (ml/h), and V2 (ml), respectively. Training procedure was replicated five times to account for the influence of the random initialization of w on the accuracy.

Accuracy of FVIII level predictions was defined as the percentage of predictions within a range of the “true” simulated FVIII level (without noise) evaluated at all simulated timepoints. This target range was set at 0.05 IU ml^−1^ for $\mu^{\text{true}}\geq 0.15$ IU ml^−1^, and at 0.02 IU ml^−1^ for $\mu^{\text{true}}<0.15$. These values represent clinically relevant differences in the FVIII level. Because patients with levels above 0.15 IU ml^−1^ hardly suffer from joint bleeding, we chose this as the lower limit.^13^ The 0.05 IU ml range represents an estimate of assay accuracy. This range was decreased to 0.02 IU ml to emphasize the importance of making accurate predictions of FVIII trough levels (e.g., <0.15 IU ml^−1^). A large difference in accuracy between the train and test set was indicative of model over‐fitting. The mean accuracy ± one standard deviation (SD) was presented for each model.

Finally, speed of the algorithm was evaluated by determining the calculation time per epoch. We calculated the gradient and updated the parameter for 100 epochs, recorded the total duration, and presented the average time spend per epoch. We used a 16 GB, Intel Core i7‐9750H CPU computer for our tests. Models were trained on the CPU only.

### Validation using real‐world data sets

Following the simulation experiment, we compared the accuracy of a priori predicted perioperative FVIII levels of a DCM and NLME model using real‐world data. Both models were developed on the retrospective dataset from Hazendonk et al.^5^ Data from the OPTI‐CLOT trial was used as an independent validation dataset.^6^ In this study, perioperative FVIII consumption was compared between PK‐guided and standard dosing regimens. FVIII levels were actively monitored and dosing was adjusted following daily measurements if required.

The one‐stage assay used in both datasets was known to significantly under‐report FVIII levels from a β‐domain deleted recombinant FVIII product (BDD‐rFVIII; moroctocog alfa/ReFacto AF).^14^ The proposed DCM architecture did not support estimation of the effect of covariates that influence the drug concentration directly. We removed all patients who received this product (9 and 4 patients in the train and validation set, respectively). The final retrospective dataset contained 110 patients with a total of 1380 perioperative FVIII measurements, and the validation set contained 62 patients with 526 measurements. Re‐estimating the NLME model parameters on the retrospective data without these patients did not lead to meaningful differences so the final model was used as is.

We fit a DCM based on patient weight, age, and having blood group O using a two‐compartment model as these covariates have generally accepted biological significance with respect to FVIII drug dynamics. We used the same $\zeta_{0}$ as in the simulation study. Additional covariates shared between the two data sets were von Willebrand factor antigen (VWF:Ag) and activity (VWF:Act) levels, hemophilia severity, and pre‐assessed surgical risk score. They were added to the base set of covariates if inclusion improved objective function value on the training data. This was somewhat similar to a stepwise procedure, although we could not use *p* values as there were no explicit parametric assumptions. Accuracy of the resulting models was evaluated on the validation set. Models were trained for 100 epochs and the set of parameters w from the epoch resulting in the highest accuracy on the retrospective data set were selected. We again performed five replications of the training procedure, resulting in five independently fit models. For the NLME model, the final model from Hazendonk et al.^5^ was implemented in NONMEM (ICON Development Solutions, version 7.4.2). Covariates used in the NLME model were patient weight, age, blood group, and surgical risk score. Accuracy was again represented as the percentage of predictions within 0.05 IU ml^−1^ of measured FVIII levels greater than or equal to 0.15 IU ml^−1^, and 0.02 IU ml^−1^ for levels <0.15.

## RESULTS

### DCM accuracy on simulated data

The accuracy of FVIII predictions by the DCM for the different scenarios is shown in Table 1. In general, a higher number of measurements or training samples resulted in improved accuracy. However, accuracy was higher for the standard DCM trained on limited measurements compared to the routine set. Slight model over‐fitting was seen when training on 20 samples but not for the other sample sizes. In all cases, we saw that initialization using $\zeta_{0}$ increased both train and test accuracy. When using initialization, there was no large improvement in accuracy when increasing the number of measurements from three (routine) to six (extensive). Furthermore, using initialization greatly improved model accuracy when only one measurement was available (from roughly 29% to 65–75%).

**TABLE 1:** Accuracy of predicted FVIII levels in the simulation experiment

Sampling strategy		Standard DCM		DCM with initialization	
	*n*	Train	Test	Train	Test
*t* = 0.5, 4, 12, 24, 36, 48	120	99.0 ± 0.21	99.1 ± 0.25	99.6 ± 0.12	99.4 ± 0.16
	60	93.3 ± 13.0	93.0 ± 12.5	98.9 ± 0.42	97.9 ± 0.18
	20	89.5 ± 1.09	84.4 ± 1.79	92.8 ± 1.76	88.7 ± 3.27
*t* = 4, 24, 48	120	65.2 ± 8.68	65.3 ± 8.86	97.8 ± 0.33	97.8 ± 0.41
	60	60.7 ± 0.61	59.5 ± 0.62	96.0 ± 0.85	94.8 ± 0.97
	20	58.2 ± 0.99	59.1 ± 0.71	96.3 ± 1.18	90.1 ± 2.00
*t* = 8, 30	120	75.9 ± 0.65	76.1 ± 1.08	90.8 ± 6.63	90.3 ± 6.19
	60	72.4 ± 1.33	73.6 ± 1.19	81.4 ± 3.29	83.0 ± 3.08
	20	66.8 ± 1.78	61.2 ± 1.41	77.7 ± 4.82	76.5 ± 2.19
*t* = 24	120	28.6 ± 3.69	28.9 ± 5.31	76.2 ± 2.74	76.0 ± 2.41
	60	29.2 ± 1.21	29.4 ± 1.02	66.8 ± 2.23	65.2 ± 2.14
	20	29.6 ± 2.68	32.2 ± 1.92	73.7 ± 1.83	72.9 ± 1.80

*Note*: Train and test accuracy is represented as the percentage of predictions within 0.05 IU ml^−1^ of true simulated FVIII levels ≥0.15 and within 0.02 IU ml^−1^ of levels <0.15. Time points are in hours. n is the number of patients in the train set. Test set size is the remainder of 500 – n. Values are represented as the mean ± one SD of five replicates.

Abbreviations: DCM, deep compartment model; FVIII, factor VIII.

**FIGURE 1:**
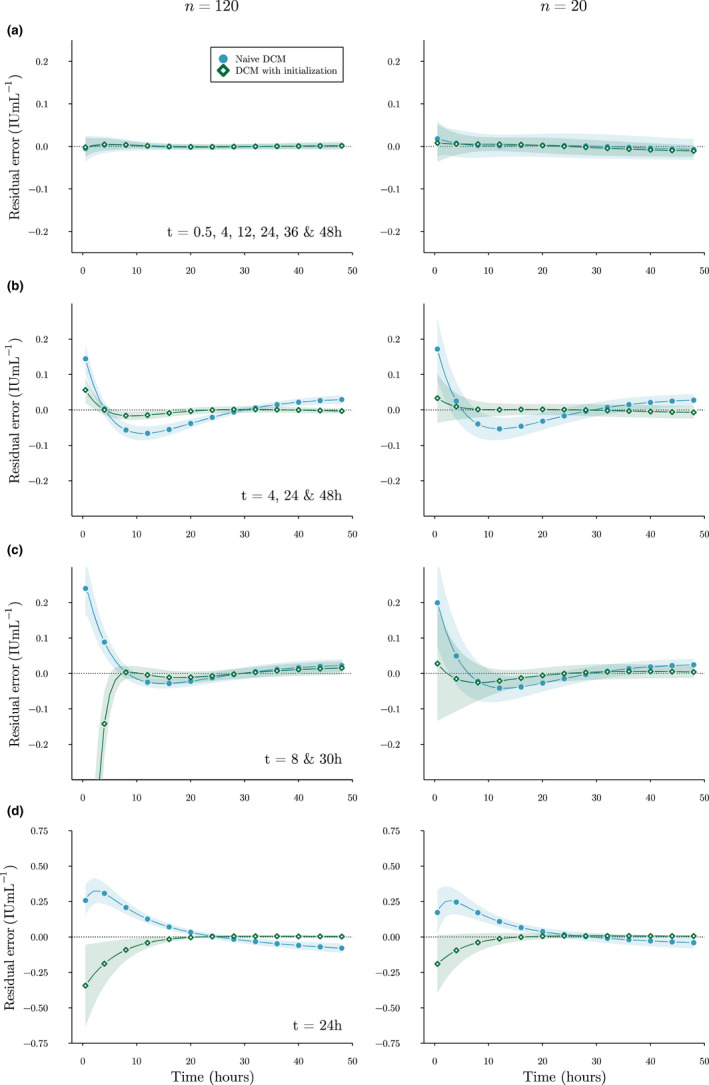
Bias and variance of residuals. Mean residuals on the test set of a single replicate of the standard DCM (circles), DCM with initialization (diamonds), and corresponding SD (shaded areas) are shown for the extensive (a), routine (b), limited (c), and extremely limited (d) sampling strategies. Points were added for the purpose of comparison. Dotted line indicates zero residual error. Images on the left were trained on 120 patients, and images on the right on 20. Positive residuals indicate underestimation of FVIII levels while negative residuals indicate overestimation. DCM, deep compartment model; FVIII, factor VIII

In Figure 1, we have depicted the mean residuals including SD for the different sampling strategies at *n* = 120 or 20. For the standard DCM, we can appreciate that decreasing the number of training samples increases variance of the residuals, whereas decreasing the number of measurements increases bias. We also see that for all but the extended measurements set high bias can be seen for peak concentration predictions. For some scenarios, using initialization is able to reduce this bias.

**FIGURE 2:**
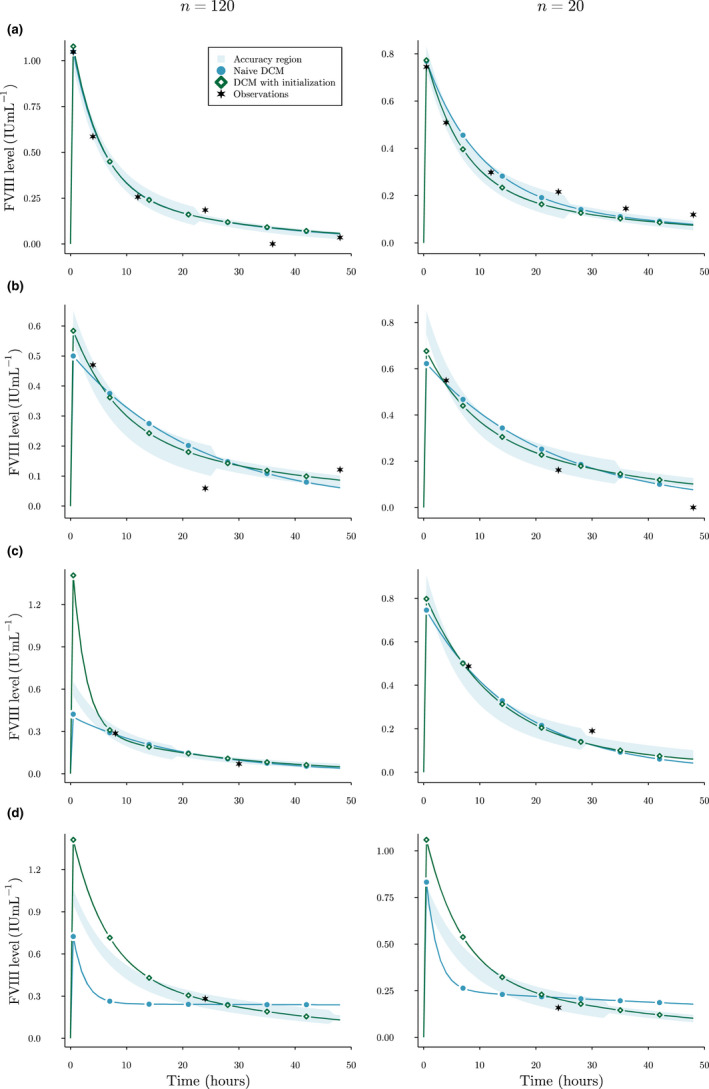
Examples of FVIII level predictions in the simulation experiment. Here, predicted FVIII levels by a single replicate of the standard DCM (circles) and DCM with initialization (diamonds) are compared. The accuracy threshold (shaded area) is also shown. Points were added for the purpose of comparison. Results are shown for a single patient for the extensive (a), routine (b), limited (c), and extremely limited (d) sampling strategies. Stars represent the observed FVIII levels. Images on the left were trained on 120 patients, and images on the right on 20. DCM, deep compartment model; FVIII, factor VIII

In Figure 2, we have shown predictions for a random patient for each of the sampling strategies. Here, we can notice that an insufficient number of measurements can allow the standard DCM to predict unrealistic FVIII responses (Figure 2d). Using initialization, we guide the DCM to find a solution that follows an initial belief about the value of each of the PK parameters.

With respect to algorithm speed, we found that time spend per epoch increased proportional to the number of samples in the train set (Table S1). The type of DCM or the number of available measurements did not affect computational time.

### Comparison with NLME model using real‐world data

In Table 2, we show the accuracy of a priori predictions of the DCM and NLME model using real‐world data. Only adding VWF:Ag to the base set of covariates resulted in an improvement of the objective function value. The DCM + VWF:Ag model showed improved accuracy on the validation set compared to the NLME model (23.1% vs. 21.6%). The base DCM had similar accuracy to the NLME model (22.0%). Time spent on training a single replicate for 100 epochs took ~ 25 s.

**TABLE 2:** Accuracy of a priori predicted FVIII levels for the independent OPTI‐CLOT data set

Model	Accuracy
NLME	21.9%
DCM	22.0 ± 0.417%
DCM + VWF:Ag	**23.1 ± 1.12%**

*Note*: Here we show the accuracy of the models as the percentage of predictions within 0.05 IU ml^−1^ of observed FVIII levels ≥0.15. For observations <0.15 this threshold was set at 0.02 IU ml^−1^. DCM accuracy is shown as the mean accuracy of five independent runs ± SD. The DCM + VWF:Ag model included VWF:Ag as an additional covariate. Bold text indicates the most accurate model.

Abbreviations: DCM, deep compartment model; FVIII, factor VIII; NLME, nonlinear mixed effect; VWF:Ag, von Willebrand factor antigen.

**FIGURE 3:**
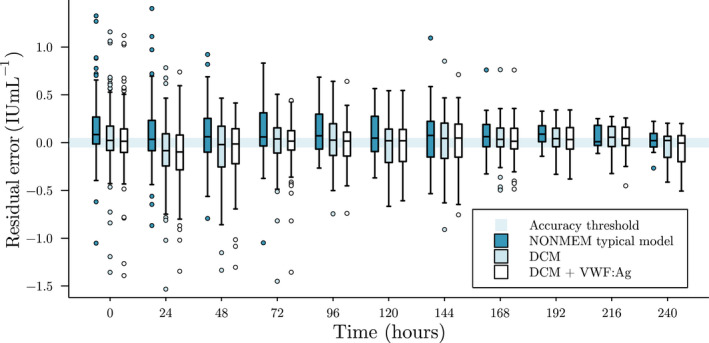
Box‐plots of residual error of predicted perioperative FVIII levels. Here, we show the residual error of a priori predictions grouped per 24 h for the NLME model (dark boxes), DCM (lightly shaded boxes), and DCM with VWF:Ag (white boxes). The shaded area indicates the 0.05 IU ml^−1^ accuracy threshold. *t* = 0 corresponds to the day of surgery. Mean prediction from the five independent DCM runs was taken to calculate residual error. Positive residuals indicate underestimation of FVIII levels, whereas negative residuals indicate overestimation. DCM, deep compartment model; FVIII, factor VIII; NONMEM, nonlinear mixed‐effect modeling; VWF:Ag, von Willebrand factor antigen

In Figure 3, the residuals of the NLME model and DCM are compared per 24 h from the day of surgery. The residual error of DCM predictions suggest lower bias, as judged by the median residual error being generally within the accuracy threshold. In contrast, the NLME model more often underestimated FVIII levels. For all models, variance of the residual error started decreasing after *t* = 72.

**FIGURE 4:**
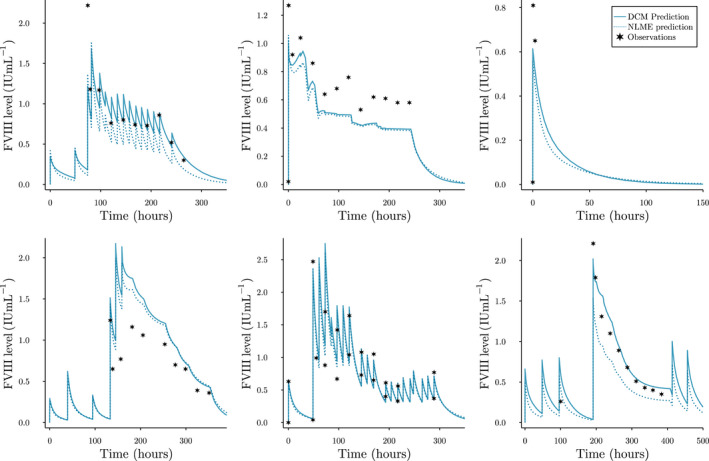
Examples of a priori perioperative predicted FVIII levels. DCM predictions represent the predicted FVIII levels by a single replicate of the DCM + VWF:Ag model. Stars represent observed FVIII levels. Both the prediction by the DCM (solid line) and the typical prediction from the NLME model (dotted line) are shown. For some patients, pre‐surgery prophylactic doses are also shown. DCM, deep compartment model; FVIII, factor VIII; NLME, nonlinear mixed‐effect modeling; VWF:Ag, von Willebrand factor antigen

In Figure 4, we have shown the prediction by the DCM + VWF:Ag compared to the NLME model for six patients. Here, we see that the DCM can accurately represent the changing FVIII levels over time when subjected to complex dosing schemes. For some patients, the DCM and NLME model predicted concentrations are very similar.

## DISCUSSION

In this study, we present a technique for improving the performance of ML models for predicting drug concentrations by constraining the solution space. Here, we have used a neural network to predict the latent parameters of a system of ODEs and determined its accuracy in different scenarios during a simulation experiment. We show that when using initialization parameters, the accuracy of such an approach is high (>80%) when training on medium‐sized datasets with at least two measurements. Next, we compared the accuracy of the DCM to an NLME model using real‐world data. The DCM displayed increased accuracy of FVIII level predictions on an independent validation set (23.1% ± 1.12 SD compared to 21.9% for the NLME model). Even though many measurements were available, achieved model accuracy was lower compared to the simulation experiment. This is indicative of the complexity of predicting perioperative FVIII levels, where other (unknown) factors seem to contribute to the IIV.

In the simulation experiment, we found that the accuracy of the standard DCM was higher for the limited sampling strategy compared to the routine sampling strategy. This suggests that it is not only the number of measurements but also their timing that can affects model bias. This is reflected in Figure 2b,c, where we can see that the routine sampling strategy leads to higher bias between t=4 and t=24 compared to the limited sampling strategy. For all scenarios, we found that using initialization parameters improved prediction accuracy. Especially when training on smaller datasets (*n* = 20), bias of residual error greatly reduced compared to a standard DCM. In small data sets, there is likely not enough data to correctly characterize the relationship between the covariates and the PK parameters. When measurements were extremely limited, a standard DCM was completely free to choose how to fit the single FVIII level and often degenerated to a flattened concentration curve (i.e., very low clearance; see Figure 2d). By using initialization, we can drive the model to follow an initial guess of compartment dynamics. However, we found that the current $\zeta_{0}$ could still lead to a biased estimation of peak concentration predictions. Similar to choosing an informative prior in the Bayesian setting, choosing the “correct” $\zeta_{0}$ can be difficult. In our case, we noticed that the DCM could maintain accurate predictions of the measurements while excessively adjusting V1. As no measurements were present at early timepoints for many of the scenarios, the model was not penalized for over or underestimating peak FVIII levels. It is thus important to choose $\zeta_{0}$ carefully by, for example, monitoring the distribution of residual errors during training and adjusting initial estimates accordingly.

The results suggest, however, that a more rigid constraint against extreme predictions is required. One such approach would be to include a prior belief over the PK parameters and performing maximum a posteriori estimation. By setting a prior distribution over our parameters we can penalize more extreme estimates. However, in the case of a neural network, this prior has to be set over the weights of each layer. Choosing a correct weight distribution that matches our prior belief over the PK parameters is very complex, and is an area of active research.^15^, ^16^ Another related improvement is the use of a Bayesian neural network.^17^ Again, using a prior over the neural network weights, we can obtain a credible interval for our parameter estimates, similar to the standard error estimates NLME produces. This allows us to contribute a measure of certainty to the PK parameters, and identify patients for which the prediction is inaccurate. It might be difficult to implement such methods relating to prior selection so other approaches might have to be evaluated.

In the real‐world experiment, the DCM trained using patient weight, age, having blood group O, and VWF:Ag achieved higher accuracy than the NLME model. Although this improvement was not extremely large, fitting and adjusting a DCM is far less time‐consuming. Training the model required only roughly 25 s, whereas development of NLME models can take far longer. A downside, however, can be that the DCM was programmed in the Julia programming language, which is unfamiliar to many pharmacometricians. We have made our model code publicly available and include a tutorial on how to fit a DCM model to any NLME compatible data set using only a few lines of code. This way, we hope to reduce the complexity of using this new technique. New covariates can simply be added to a base set of covariates and accuracy can be monitored during training. The method also allows for the user to simulate new treatment strategies by adjusting $I_{i}$. As seen in Figure 4, the model accurately represents the changing concentration over time.

We have shown examples where we use a DCM to estimate the effect of all covariates, but it is also possible to add a layer where the relationship between a covariate and the PK parameters is explicitly stated. An example would be to use allometric scaling to represent the effect of weight on the PK parameters, while having the neural network learn the effect of the other covariates using standard layers. The practical use of this concept will have to be evaluated.

From the above experiments some limitations of the DCM have come to light. First, it is sometimes the case that no prior knowledge exists for choosing an appropriate compartment model to describe the drug concentrations. In these cases, we suggest fitting multiple DCM models with different model structures and inspect the solution in order to resolve model misspecification. Next, the proposed architecture of the DCM does not support covariates that affect the predicted concentration directly. This has resulted in the removal of all patients in the datasets who received BDD‐rFVIII. In the NLME model, this effect can be directly estimated in the model, whereas for the DCM estimating this quantity next to w can be difficult. The DCM also does not quantify any form of residual variability. Use of the MSE implicitly assumes simple additive error, where in many cases a combined additive and proportional error model is more appropriate. In addition, the model does also not quantify residual IIV, making the model potentially more susceptible to over‐fitting. We have performed some prior work on combining the DCM with the extended least squares objective function as a possible solution to these problems.^18^ We, however, found that the implementation is unstable and requires careful tuning of training parameters. More work is required to improve the random effect estimation when using neural networks. Finally, although the relationships between PK parameters and covariates can be visualized after fitting the DCM, understanding the relationships between covariates and PK parameters can be difficult. ML explanation methods, such as SHAP,^19^ can be performed in order to help visualize these relationships. Fact remains that neural networks are black box models, and the discussion of trust in ML method in the field of pharmacometrics is still in its infancy.

In conclusion, the DCM is a reliable tool for introducing ML models in population PK analysis. The DCM can automatically learn covariate relationships from data reducing the need for tedious covariate analysis. In contrast to other ML models, the DCM is based on compartment models allowing for the implementation of prior knowledge of drug dynamics. In addition, the DCM can be used with any dosing scheme, and allows for reliable extrapolation to different timepoints.

## AUTHOR CONTRIBUTIONS

A.J., F.W.L., M.H.C., and R.A.A.M. wrote the manuscript. A.J. and R.A.A.M. designed the research. A.J. performed the research. A.J. analyzed the data.

## CONFLICT OF INTEREST

The authors declared no competing interests for this work.

## Supporting information


Table S1
