# Supplementary material for: Deep compartment models: A deep learning approach for the reliable prediction of time‐series data in pharmacokinetic modeling
Source: CPT Pharmacometrics Syst Pharmacol. 2022 May 27;11(7):934–45. doi: 10.1002/psp4.12808 (PMC9286722; doi:10.1002/psp4.12808)
Supplement: Supplementary file 1 — Table S1 [file PSP4-11-934-s001.docx]

Table S1. Time spend per epoch in the simulation experiment.

*Time per epoch (seconds) represents the average time spend on gradient calculation and parameter update when training for 100 epochs.*

| Sampling strategy | $n$ | Time per epoch Standard DCM (seconds) | Time per epoch  DCM with initialization (seconds) |
| --- | --- | --- | --- |
| $t=$ 0.5, 4, 12, 24, 36, 48 | 120 | 0.083 | 0.083 |
|  | 60 | 0.046 | 0.042 |
|  | 20 | 0.015 | 0.015 |
| $t=$ 24 | 120 | 0.091 | 0.084 |
|  | 60 | 0.043 | 0.042 |
|  | 20 | 0.016 | 0.014 |
